# Evidence-Based Annotation of the Malaria Parasite's Genome Using Comparative Expression Profiling

**DOI:** 10.1371/journal.pone.0001570

**Published:** 2008-02-13

**Authors:** Yingyao Zhou, Vandana Ramachandran, Kota Arun Kumar, Scott Westenberger, Phillippe Refour, Bin Zhou, Fengwu Li, Jason A. Young, Kaisheng Chen, David Plouffe, Kerstin Henson, Victor Nussenzweig, Jane Carlton, Joseph M. Vinetz, Manoj T. Duraisingh, Elizabeth A. Winzeler

**Affiliations:** 1 Genomics Institute of the Novartis Research Foundation, San Diego, California, United States of America; 2 Department of Cell Biology ICND202, The Scripps Research Institute, La Jolla, California, United States of America; 3 Department of Pathology, New York University, New York, New York, United States of America; 4 Department of Infectious Diseases and Immunology, Harvard School of Public Health, Boston, Massachusetts, United States of America; 5 Division of Infectious Diseases, Department of Medicine, University of California San Diego, San Diego, California, United States of America; Harvard School of Public Health, United States of America

## Abstract

A fundamental problem in systems biology and whole genome sequence analysis is how to infer functions for the many uncharacterized proteins that are identified, whether they are conserved across organisms of different phyla or are phylum-specific. This problem is especially acute in pathogens, such as malaria parasites, where genetic and biochemical investigations are likely to be more difficult. Here we perform comparative expression analysis on *Plasmodium* parasite life cycle data derived from *P. falciparum* blood, sporozoite, zygote and ookinete stages, and *P. yoelii* mosquito oocyst and salivary gland sporozoites, blood and liver stages and show that type II fatty acid biosynthesis genes are upregulated in liver and insect stages relative to asexual blood stages. We also show that some universally uncharacterized genes with orthologs in *Plasmodium* species, *Saccharomyces cerevisiae* and humans show coordinated transcription patterns in large collections of human and yeast expression data and that the function of the uncharacterized genes can sometimes be predicted based on the expression patterns across these diverse organisms. We also use a comprehensive and unbiased literature mining method to predict which uncharacterized parasite-specific genes are likely to have roles in processes such as gliding motility, host-cell interactions, sporozoite stage, or rhoptry function. These analyses, together with protein-protein interaction data, provide probabilistic models that predict the function of 926 uncharacterized malaria genes and also suggest that malaria parasites may provide a simple model system for the study of some human processes. These data also provide a foundation for further studies of transcriptional regulation in malaria parasites.

## Introduction

Drug targets for many pathogenic microbes such as *Mycobacterium tuberculosis* or *Plasmodium falciparum* are often selected from proteins identified through genome sequencing efforts, for which enzymatic function is already known allowing for biochemical assay development in drug screening. This expedient approach means that many genes encoding proteins with uncharacterized functions may be disregarded as potential therapeutic or disease-preventive targets. These assumptions have several consequences. First, the diversity in many chemical libraries is not utilized. Second, focusing on the pathogen enzymes most likely to be essential to all life increases the likelihood that a compound will also have activity against the host.

While using well-characterized targets (e.g. dihydrofolate reductase, dihydropteroate synthase, ubiquinol-cytochrome c reductase) studied previously in model systems such as *Saccharomyces cerevisiae* or *Escherichia coli* is perhaps inevitable for practical reasons, the genome sequence of the malaria parasite, *P. falciparum*, does encode a plethora of potentially novel drug and vaccine targets as indicated in the genome sequencing project [1]. It has also been noted that the proportion of uncharacterized genes *in P. falciparum* is much higher than in many other sequenced genomes [1]. While some of these uncharacterized proteins may be very specific to *P. falciparum*, which causes the most severe form of human malaria, others have orthologs in many other parasites and even in plants, but their roles are not understood in any species. Apicomplexan parasites, including *Babesia*, *Theileria*, *Toxoplasma*, and *Cryptosporidium spp.* generally are not easy to manipulate experimentally as many have complex life cycles within multiple hosts and it may be difficult to obtain sufficient quantities of parasites for biochemical assays. Given the impact of malaria on human society, the problem of discovering what these conserved, but uncharacterized, genes are doing for the cell is particularly pressing and new approaches that take advantage of empirical data derived from parasites instead of model systems are needed. Here we use detailed cross-species expression data to create maps that associate uncharacterized genes with different cellular processes with an emphasis on those that are not represented in model systems.

While numerous microarray or cDNA sequencing experiments have been performed on malaria parasites, the use of two-channel comparative formats, different array designs and, in particular, the limited coverage (e.g. 2,045 independent clones obtained from a *P. berghei* mung bean nuclease DNA libraries) sometimes limits the usefulness of these data. To illustrate, a cDNA library was constructed from liver stage schizonts that had been obtained by laser capture microdissection and 623 transcripts were identified [2]. Of these, 25% were not detected by cDNA sequencing of blood or sporozoite stages. However, inspection shows some of the presumed liver-specific transcripts are likely the result of sequencing an insufficient number of cDNAs in the different stages. Replication factor A is reported as being expressed exclusively in liver stages although there is only one copy of this gene in the genome and DNA replication is also happening in blood and oocyst stages. Likewise, GMP synthetase is reported as expressed exclusively in liver stages while knowledge of metabolism suggests that its product should be required throughout the lifecycle. Indeed, previous transcription and proteomic studies of *P. falciparum* indicate high levels of GMP synthetase in trophozoite stages. PY01200, a transcript shown to be liver specific by RT-PCR also shows high expression in *P. falciparum* merozoites and protein has been detected in *P. falciparum* sporozoites. A very comprehensive expression analysis of *P. falciparum* blood stages [3] was also performed, but again, without considering insect stage data, it is hard to determine the significance of genes showing low amplitude changes in the erythrocytic cycle. These observations do not indicate that the data from these previous studies are of poor quality, but rather that coverage is insufficient to assume presence and absence of transcripts in the various parasite lifecycle stages. Proteomic expression data is also available for many parasite species [4], [5], [6], [7] but coverage, especially when only high quality spectra are used, is generally much sparser than for microarray studies, thus limiting their usefulness. For example, one would expect stochiometric representation of proteins comprising different protein complexes (e.g. proteosomes, ribosomes, t-complex) across parasite lifecycle stages in proteomic data from *P. berghei* but this is not observed due to random sampling error at low coverage and chance. Likewise, histones, a component of chromatin that should be found in all lifecycle stages, are detected in only some stages—H3 is not found in ookinetes while H2A is not found in sporozoites [7]. Thus, more studies are necessary. Here we aim to provide the first comprehensive genome-wide expression analysis of malaria parasite transcription including all life cycle stages.

Even if comprehensive data were already available for malaria parasites, a robust method for analyzing all of the data would also be needed. Researchers generally use gene expression data to find new genes involved in processes using a “guilt-by-association” approach and to understand transcriptional regulation. In both cases genes first need to be clustered by their expression similarity. However, cluster boundary determinations often are subjective and non-optimal for the purpose of function prediction. This challenge is addressed by an algorithm termed Ontology-based Pattern Identification (OPI) [8], which has been shown to identify gene clusters of better quality than unsupervised clustering algorithms such as the robust *k*-means clustering we used previously [9]. The OPI analysis begins with a piece of knowledge, such as a group of genes believed to share a set of characteristics—e.g. all genes known to be involved in gametocytogenesis in malaria parasites. From this list of “seed genes” an “average” expression profile that best represents gametocytogenesis can be constructed, and all of the malaria genes in the genome can be relatively ranked according to their similarity to the average profile as measured by Pearson correlation. Genes ranked near the top are more likely to be involved with the gametocytogenesis process. OPI iteratively descends the rank list and identifies a cutoff, where the largest number of seed genes are included within the smallest cluster size as computed by the minimization of a hypergeometric probability score. As the *p*-value represents the odds of non-seed genes in the resultant cluster sharing a similar expression profile to the seed genes by chance, OPI clusters automatically balance between false discovery rate and true positive rate for the purpose of functional prediction, without relying on subjective parameters. This process is repeated for all possible pieces of knowledge and results in gene clusters containing both known genes and unassigned genes for each functional group. The resultant clusters are then subjected to statistical permutation tests. It is apparent that the accuracy of such predictions relies on both the completeness of the underlying data set and the quality of the seeding knowledge. Although we had previously carried out OPI studies, it was based on a limited set of *P. falciparum* data, as well as knowledge obtained from the Gene Ontology (GO) consortium [10], which may not represent parasite-specific processes well. The accuracy of our predictions in this study is significantly improved as we not only combine gene expression data from both *P. yoelii* and *P. falciparum* and cover virtually all lifecycle stages, but also establish a systematic literature mining pipeline to explore many new groups of functionally-related genes based on co-citation in 1,278 malaria-related publications. To validate the predictions we apply the same routine to human and yeast expression data, as well as protein-protein interaction data, and illustrate that the method can accurately predict many functions. The results are a searchable database that can be used by researchers seeking information about the possible functions of uncharacterized malaria proteins as well as a comprehensive set of unprocessed data that may be of use to those interested in systems biology.

## Results

### *P. yoelii* sporozoite transcription

To infer function of malaria proteins, we had previously collected detailed expression data from *P. falciparum* [9]. Here we add *P. falciparum in vitro* zygote and ookinete gene expression data, as well as data from a variety of developmental stages from the rodent malaria parasite, *P. yoelii*. For this project, rodent parasite data were collected from ∼150,000 25mer *P. yoelii* probes contained on a previously described custom-designed oligonucleotide array [9]. These new *P. yoelii* data included oocyst-stage sporozoites, blood-stage parasites, late-stage gametocytes and 36 and 40 hour liver stage parasites obtained by a cDNA subtraction method. Previously described *P. yoelii* salivary gland sporozoite data were also used [11]. Expression data were collected for 5,521 different *P. yoelii* genes. The new expression data for *P. yoelii* sporozoite stages agree with results obtained previously from sequencing cDNA libraries [12]. As expected, *UIS3* (Up In Sporozoites) shows substantial (5-6 fold) induction in salivary gland sporozoites relative to oocyst stage sporozoites [13]. Interestingly, although *UIS3* is transcribed in sporozoites, it has been shown to be essential for liver stage development, [14] suggesting that transcriptional silencing may also play a role in the biology of exoerythrocytic forms (EEFs) of malaria parasites. Likewise, *UIS4* (PY00204) is upregulated. This gene has a *P. falciparum* homolog (PF10_0164, by syntenic analysis) that is abundantly expressed in sporozoites and encodes a member of the early transmembrane protein family important for establishing the parasitophorous vacuoles in blood stage parasites [15]. The rodent version is also critical for early liver stage development rather than sporozoites function [16]. Other proteins with similar expression patterns (i.e., induction in salivary gland sporozoites relative to oocyst stages) are likely to be essential for early liver stage development. Genes highly expressed in midgut sporozoites relative to salivary gland sporozoites are mostly uncharacterized but include MAEBL (PY05977) and a calcium dependent kinase (PY06394), both of which were upregulated by about 20 fold.

### *P. falciparum* ookinete and zygote transcriptional patterns

New *P. falciparum* ookinete and zygote data indicate unexpected dynamics of gene expression that likely reflect novel mechanisms of gene expression regulation including post-transcriptional repression [17]. The zygote/ookinete stages of *P. falciparum* are diploid and *P. falciparum* ookinetes have been difficult to isolate in large numbers. Here, we were able to obtain *P. falciparum* ookinetes using a modified approach [18] in which an estimated 5–10% morphological transformation of zygotes to ookinetes was seen. The genes showing the largest expression values in ookinetes included many glycolysis genes such as glyceraldehyde-3-phosphate dehydrogenase and fructose-bisphosphate aldolase, which are not highly transcribed during gametocytogenesis, reflecting a metabolic shift needed for development within the mosquito midgut. The most highly expressed genes in the zygote (with 6 or more probes) included the heat shock 70 protein, PF08_0054 (ranked 1st); the *P. falciparum* gamete antigen, PF13_0011 (ranked 2nd); a sexual stage specific protein, PFD0310w (ranked 24th); and circumsporozoite-trap related protein (CTRP), PFC0640w (ranked 20th). Many of the most highly transcribed genes with rodent homologs had been identified in analysis of the rodent ookinete proteome at levels that would not be expected by chance. Among genes with expression values of greater than 1000 (≥6 probes), and with rodent orthologs (233 genes), 96 were detected with more than 4 spectra in the ookinete proteome analysis. In contrast, only 15 genes with more than four spectra were detected amongst the 270 with transcript levels of less than 25. This difference is not expected by chance (*p* = 10^−21^ by χ^2^-test). Several of the most abundant genes in the proteome analysis were also the most abundant as measured on the array including a glyceraldehyde-3-phosphate dehydrogenase (ranked 3rd by proteomics, 2nd by gene expression) and heat shock protein 70 (ranked 5th by proteomics, 3rd by gene expression, out of 3,137 genes with 6 or more probes). The 15 proteins that were detected by MudPIT but were not considered “transcribed” included a helicase, and a translation factor. It is difficult to determine whether these represent species-specific differences, annotation problems or the effects of translational repression [7], [17], [19], because in most cases transcripts were detected in earlier gametocyte stages. Genes considered abundant by transcript analysis, but which were not detected in the proteomic analysis included many membrane proteins, which can be difficult to detect by mass-spectrometry. Several highly expressed genes do not have rodent orthologs and thus their abundant expression has not been previously reported. Although *P. falciparum* ookinetes and zygotes are morphologically similar, comparison of gene expression patterns showed that a clear up- and down-regulation of some genes was occurring, despite an overall similarity (*r* = 0.92). Differences between the zygote and the ookinete included a 5-fold downregulation of the transcript for chitinase, a gene required for penetration of the mosquito midgut epithelium, which is likely transcribed in zygotes and used in ookinetes [20], and a 5-fold downregulation of PFC0640w, the CTRP [21], a gene required for gliding motility in ookinetes. The downregulation of important ookinete proteins mostly likely reflects the kinetic differences between transcription and translation [19], [20].

### *P. yoelii* liver stage transcription

We also examined expression data from *P. yoelii* liver stage parasites. From an immunological perspective this is the most interesting stage but also the most difficult to study. We performed a cDNA subtraction using RNA from uninfected livers because of the high potential for host RNA contamination. We anticipated that this would be necessary to detect parasite transcript signal because the parasite genome is 1,000 times smaller than the mouse genome and because only 10% of the cells were infected. Overall, these data were of a lower intensity than for other samples, but the expression differences relative to blood stages and the enrichment of genes involved in various pathways were consistently observed between the two liver samples. The characterized genes (Table 1) that showed the largest rank ratio changes in liver against mixed blood stages in *P. yoelii* were PY01852, a putative phosphotidylethanolamine binding protein; PY03168, the circumsporozoite protein; PY00446, a polyprenyl synthetase; PY00573 lipoamide dehydrogenase; and PY01586, beta-hydroxyacyl-acp dehydratase precursor. With the exception of the circumsporozoite protein, which is translocated to the host hepatocyte cytoplasm where it exerts a profound effect on host transcription [22], the rest are localized to the apicoplast [23]. The apicoplast, like the chloroplast, is a thought to be a remnant of an endosymbiotic bacterium and is the site of type II fatty acid biosynthesis. Several other proteins with non-specific roles in signaling were also upregulated including PY01269, a phosphotyrosyl phosphatase activator; PY01857, a cAMP-specific 3′, 5′-cyclic phosphodiesterase; and PY07390, a calcium-dependent protein kinase. Genes showing the greatest upregulation in blood stages relative to liver included many ribosomal proteins, plasmepsins, heat shock proteins, glycolysis enzymes (e.g. lactate dehydrogenase) and a falcipain. The plasmepsins and falcipains are proteases involved in hemoglobin degradation [24] and thus their absence in the liver stages is to be expected.

**Table 1:** Genes upregulated 36 and 40 hpi in liver stages relative to blood stages.

*P.f.* gene	*P. y.* gene	Function	Rank	Description
PF14_0280	PY01269	Signaling	6, 110	Phosphotyrosyl phosphatase activator
PFL0955c	PY01852	Fatty-acid metabolism	8, 9	Phosphotidylethanolamine-binding protein
PFC0210c	PY03168	Sporozoite	14, 7	Circumsporozoite (CS) protein
PFB0130w	PY00446	AP-localized fatty acid biosynthesis	40, 120	Polyprenyl synthetase
PF08_0066	PY00573	AP-localized fatty acid biosynthesis	47, 19	Lipoamide dehydrogenase
MAL13P1.119	PY01857	Purine metabolism	39,88	CAMP-specific 3′,5′-cyclic phosphodiesterase 4B
PFL0475w	PY01829	Purine metabolism	38, 103	3′,5′-cyclic-nucleotide phosphodiesterase
PF14_0227	PY07390	Signaling	85,31	Calcium-dependent protein kinase
PFL1260w	PY03530	Folate biosynthesis	45, 69	Hydrolase/phosphatase
PF11_0117	PY01741	DNA replication	84, 222	Replication factor C subunit 5
PF10_0330	PY00468	Signaling	122, 49	Ubiquitin-conjugating enzyme
PFL2250c	PY00403	Signaling	78, 32	Rac-beta serine/threonine protein kinase
MAL13P1.95	PY03801	Iron metabolism	66, 374	Ferredoxin
PFD0825c	PY04369	Translation	93, 565	RNA-binding protein of pumilio/mpt5 family
PFD0215c	PY01340	Sporozoite	60, 119	*P. Berghei* pbs36-related
PFL2460w	PY01337	Cytoskeleton	62, 266	Coronin
PFC0831w	PY00756	AP-localized fatty acid biosynthesis	130, 76	Triosephophate isomerase
PFD0260c	PY04387	Adhesion to CD36 [66]	123, 1	Sequestrin
PFL2510w	PY00008	Sporozoite	120, 155	Chitinase
PFE0175c	PY00345	Sporozoite	184, 278	Unconventional myosin pfm-b
PFL2210w	PY05459	Porphyrin metabolism	208, 483	delta-aminolevulinic acid synthetase
PF13_0128	PY01586	AP-localized fatty acid biosynthesis	91,26	Beta-hydroxyacyl-acp dehydratase
PFB0325c	PY02063	Sporozoite	110, 154	Cysteine protease
MAL13P1.190	PY02721	Protein degradation	107, 258	Proteasome regulatory component
PFI0955w	PY05332	Transport	89, 28	Sugar transporter

The rank indicates the results from two independent cDNA subtraction experiments. Genes described as “hypothetical” are not listed. The rank is derived from comparing levels in *P. yoelii* asexual stages to liver stages and is calculated only for genes of at least six probes and changing more than 3-fold within the examined conditions (54 for 3,450 *P. falciparum* and *P. yoelii* gene pairs total). Genes listed “sporozoite” are highly expressed in salivary gland sporozoites, and their continued detection may be the result of some sporozoites failing to develop, or may indicate that there is a continued need for the gene product in the liver, potentially because the product is involved in immune response modulation.

### Grouping genes by expression pattern and statistically testing associations

To create models for the function of various malaria genes and to further validate the data, *yoelii-falciparum* ortholog pairs were identified and the combined expression data vectors were then hierarchically arranged. The OPI clustering algorithm was then applied, which relies on knowledge such as Gene Ontology annotations to optimize cluster boundaries, normalization and weighting methods, so that the highest proportion of genes with particular classifications is contained in the smallest sized cluster of co-expressed genes. The approach allows us to assess the quality of the expression data and is more rigorous than spot-checking a random collection of genes. For example, it is now accepted that genes encoding proteins that are part of multi-protein complexes (e. g. ribosomes) are likely to be co-transcribed. Therefore, if expression data are universally high-quality, one would expect that almost all members of a complex to show highly correlated expression patterns across conditions and that the correlation would be much higher than if genes were randomly assorted. While it is relatively trivial to choose a few genes and then to show that they are upregulated by quantitative RT-PCR, or that their proteins are developmentally regulated by a western blot, such an approach just validates the behavior of the genes that were chosen (and whose selection might not have been random) and does not reveal much about the quality of the measurements for the other 5000 genes in the dataset. However, there are also problems with relying solely on statistical measurements because of inherent correlations between genes and ontology terms. Therefore, permutation testing was performed.

**Figure 1:**
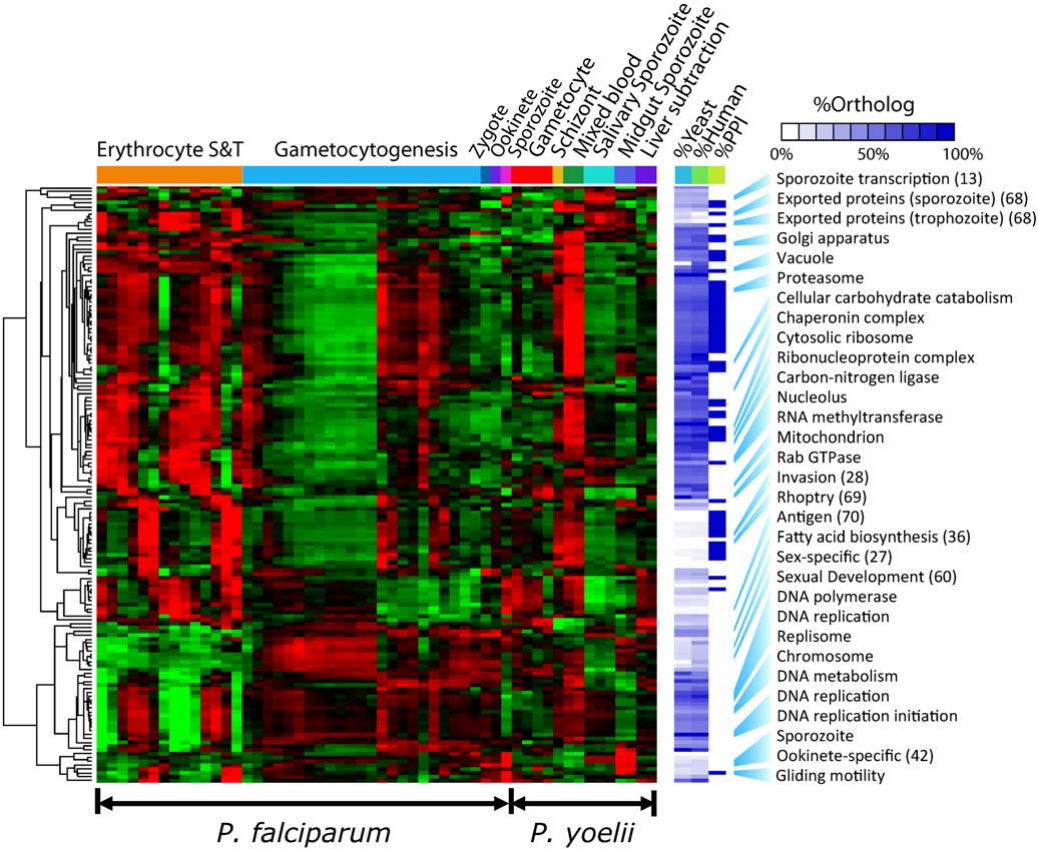
Temporal expression patterns were constructed from 54 *P. falciparum* and *P. yoelii* life cycle samples. A total of 156 statistically enriched gene clusters identified by OPI analysis illustrates the transcription regulation characteristics of all key biological processes in *Plasmodium* species. Their yeast and human orthologs contents are represented by the white-blue heatmap, indicating parasite-specific processes generally found fewer orthologs in model organisms. The percentage of proteins that form statistically significant within-cluster networks are also white-blue color coded; most networks occur in blood stage processes. Altogether 33 manuscripts were identified with significant overlap to the clusters, nine of which [13], [27], [28], [36], [42], [60], [68], [69], [70] are referenced in the figure. Two clusters were enriched for proteins predicted to have a parasite export signal [68] and were labeled as “Exported proteins (sporozoite)” and “Exported proteins (trophozoite)”—one of which peaks in the trophozoite stage and a second which peaks in sporozoite stages (see GO:PM15591202_Trp and GO:PM15591202_Spo in Table S1-S2). S & T indicate that the *P. falciparum* parasites were synchronized within the asexual cycle by the thermocycling or sorbitol method [9]. The figure does not comprehensively describe all gene expression patterns contained within the data as there are ∼1,026 genes which are not found in any of the groups depicted here because they do not share expression patterns with a sufficient number of previously characterized genes.

Applying OPI resulted in 98 non-redundant clusters derived from gene ontologies highly enriched for processes such as glycolysis or protein synthesis, or for cellular components such as the proteosome core complex (Figure 1, Table S1). While we had previously performed a similar analysis on a limited set of sexual development and erythrocytic stage *P. falciparum* data, the addition of the new data from oocyst and salivary gland sporozoite stages and the creation of combined expression vectors with data from both human and rodent parasites substantially improved the quality of the predictions and allowed the separation of genes which had previously been grouped together. In a previous analysis of sexual development and asexual cycles we identified 246 genes associated with gametocytogenesis, which included the genes involved in type II fatty acid biosynthesis such as PF11_0256, the pyruvate dehydrogenase E1 component. Here we can show that while type II fatty acid biosynthesis genes are upregulated during sexual development they are also upregulated in liver stage development, while others are not. For this study the *p*-values for functional enrichment calculated with the accumulated hypergeometric distribution ranged from 10^−69.0^ to 10^−8.1^. An example of one of the clusters is shown in Table 2 (others can be downloaded as additional data files, http://carrier.gnf.org/publications/Py, the companion website), which shows the *P. falciparum* genes in a group enriched for the cellular component, nucleolus (GO:0005730). This group contains eight of the fourteen annotated *P. falciparum* or *P. yoelii* nucleolus genes in a group of 28. Given that 6,592 genes were considered in this analysis the probability of enrichment by chance is very low (*p* = 10^−15.3^). Not only is the *p*-value low, but it is likely much higher than it should be: Evidence compiled independently indicates that almost every “hypothetical” gene in the cluster has a yeast ortholog, most with likely roles in RNA polymerase I processing and transcription (Table 2). There are numerous other similar examples of the quality of the expression data as evidenced by the functional enrichments. For example, of the twelve genes in GO group GO:0005663 (DNA replication factor C complex), twelve are found in a cluster of 27 (*p* = 10^−28.9^), with most of the other genes having a role in DNA replication. Of the 12 components of the chaperonin-containing T complex (GO:0005832), ten are contained in a group of 12 genes (*p* = 10^−27.2^). The gluconeogenesis cluster contains 9 of the 13 annotated genes in a group of 14 (*p* = 10^−21.9^), with lactate dehydrogenase considered a miss. The patterns of gene regulation for the different functionally-enriched categories can be seen by following the “OPI Web Portal” link on the companion website and the representative profile for each cluster is shown in Figure 1.

**Table 2:** Example of a gene expression cluster enriched for genes with roles in RNA polymerase nucleolus I activity (GO:0005730).

*P.f.* name	InGO	Yeast name	Yeast function	Current *P.f.* annotation
PF11_0305	Y	YNL061W	Probable RNA m(5)C methyltransferase, essential for processing and maturation of 27S pre-rRNA and large ribosomal subunit biogenesis	hypothetical protein
PF11_0358	Y	YPR010C	RNA polymerase	RNA polymerase, beta subunit
PF11_0471	N	YCR072C	WD-repeat protein involved in ribosome biogenesis	hypothetical protein
PF13_0219	N	YMR229C§	Protein required for the synthesis of both 18S and 5.8S rRNA	hypothetical protein
PF07_0121	N	YHR170w	Protein involved in nuclear export of the large ribosomal subunit	hypothetical protein
PF10_0200	N	YNL132w	ribosome biogenesis and assembly	hypothetical protein
PF13_0165	N		Required for 18S rRNA and 40S ribosomal subunit production in Schizosaccharomyces pombe [67]	multidomain scavenger receptor protein PbSR precursor
PF08_0055	Y	YNL175w	u3 small nucleolar ribonucleoprotein protein, putative	hypothetical protein
PF11_0090	N	YGR103w	NOP7	hypothetical protein
PF14_0734	N			hypothetical
PFD1175w	N	YMR173w	Interacts with ribosomal proteins by two hybrid	hypothetical protein
PF14_0677	N	YOL010W	RNA terminal phosphate cyclase-like protein involved in rRNA processing at sites A0, A1, and A2	RNA 3′-Terminal Phosphate Cyclase-like protein
PFE0465c	Y	YOR341W	RNA polymerase I;	RNA polymerase I
PFI1040c	N	YLR101C§	Dubious ORF	hypothetical protein
PFL1345c	N	YPL086C	Subunit of Elongator complex, which is required for modification of wobble nucleosides in tRNA	hypothetical protein
PF11_0305	Y	YNL061W	Probable RNA m(5)C methyltransferase, essential for processing and maturation of 27S pre-rRNA and large ribosomal subunit biogenesis	hypothetical protein
PF11_0358	Y	YPR010C	RNA polymerase	RNA polymerase, beta subunit
PF11_0471	N	YCR072C	WD-repeat protein involved in ribosome biogenesis	hypothetical protein

For brevity, only *P. falciparum* genes (15 of 28 total) are shown. The heading “InGO” indicates whether or not the gene was annotated as being part of the GO category and used to determine cluster boundaries. Yeast genes indicated with a § were matched by BLAST searching the *P. falciparum* or *P. yoelii* protein against the *Saccharomyces cerevisiae* translated genome and selecting the best scoring hit, a necessity as many of these genes are small. YLR101C is antisense to an essential gene and is considered “dubious.”

One drawback of expression-based function assignment is that in some cases different processes may share similar expression patterns due to possible incomplete coverage of lifecycle stages, inherent noise in the measurements, or the possibility that transcripts for proteins with relatively different cellular roles may be controlled by the same transcription factors. For example, genes involved in both ribosomal protein function and glycolysis are both transcriptionally induced during the trophozoite, or feeding stage of parasite growth. We thus also calculated a false discovery rate (FDR) for each group of genes. Smaller clusters often have FDR below 50%, indicating its function assignments are of better quality compared to larger clusters of higher FDR, therefore only clusters of size ≤500 were reported (Figure 1, Table S1, S2). The FDR assigned here tend to be conservative, because false discovery (FD) genes in some clusters are likely due to incorrect or incomplete annotation. For example, a group 16 genes enriched for proteins with roles in DNA replication initiation (GO:0006270) contains ten of the 15 annotated genes (*p* = 10^−23.4^). Examination of the list, however, shows that four of the six “FD genes” are DNA replication licensing factors, proteins needed for DNA replication initiation.

### Validating the data with *S. cerevisiae* expression patterns

Clusters with a low FDR contain mostly known proteins, and therefore one can postulate a role for many conserved FD proteins using annotations and the literature for these clusters. However, to identify correct predictions for more difficult clusters, we examined a collection of *S. cerevisiae* gene expression data that had been collected on the S98 array and human expression data derived from 79 tissues collected on the U133A array [25]. These data were processed in a manner similar to the *Plasmodium* gene expression data resulting in 1,046 and 684 statistically significant groups, respectively. For each *Plasmodium* gene in an OPI cluster, we looked for the occurrence of its yeast and human orthologs in the related GO clusters in model organisms as additional evidence of correct functional assignment. To be conservative, only evidence from the same or child cross-species GO clusters were automatically compiled, which resulted in 2,807 gene-GO assignments for 696 malaria genes (Table S2). These assignments included all co-expression evidence, i.e., assignments supported by either yeast or human co-expression analysis, as well as those derived from clusters identified based on literature mining approach as discussed later. There are numerous examples of cross-species support for uncharacterized proteins, in fact, as many as 66% (1,839) of our functional assignments have not been captured by existing manual curation. The OPI cluster generated using the replisome GO group (GO:0030894) contains 22 of 30 members of the replisome in a group of 109 genes. This cluster contains PFE0090w and PFF0785w, uncharacterized proteins whose orthologs are associated with the numerous DNA replication processes in humans and yeast (e.g. yeast replisome, *p* = 10^−9.38^, human alpha DNA polymerase primase complex, *p* = 10^−5.31^). Most of the proteins in this group have expression profiles that peak while DNA replication is occurring in blood, liver and sexual stages and appear to have roles in cell cycle processes. Another gene that has strong support for its involvement in translation is PF08_0019 (yeast ortholog, YMR116C). This gene is found in a cluster that is strongly enriched for components of the eukaryotic cytosolic ribosome (GO:0005830) in *Plasmodium* species (*p* = 10^−37.2^) and *S. cerevisiae* (p<10^−163.0^). Furthermore, while not annotated as being a component of the ribosome, its disruption in *S. cerevisae* leads to defects in translation [26]. Of course, these data are dependent on having accurate gene models and protein sequence alignments. If the protein sequence is not correctly predicted, spurious matches between unrelated genes may occur. Indeed, based on our work expressing recombinant proteins we estimate that there may be minor problems with the gene models for 20% of *P. falciparum* proteins. These expression data may assist in correcting and evaluating gene models.

### Confirmation in human expression patterns

Better support for the functions of proteins was sometimes found in the human gene expression atlas data compared to the yeast data. One striking category was the group of genes induced during gametocytogenesis in *Plasmodium spp*. (GO:GNF0004). Malaria parasites form male and female gametes within the mosquito and *Plasmodium spp.* male gametes, like their human counterparts, bear flagella. A number of the genes in this cluster of 480 genes have human orthologs, but as one might expect, no yeast orthologs. Of these genes 26 are found in a spermatogenesis-specific human cluster (483 transcripts, *p* = 10^−28.2^). The gene PF13_0269 encodes a putative glycerol kinase and is induced during gametocytogenesis. Its human ortholog, GK2, is strongly associated with male sexual development with a large induction of expression specifically in human testis-derived tissues. While the co-expression of characterized genes forming the flagellar axoneme is expected based on previous work [27], the presence of uncharacterized *P. falciparum* proteins, PFL0325w, PF14_0493, PFL1295w, and PF13_0060, all of which map to uncharacterized genes in the human spermatogenesis cluster, was not expected. We also found that some genes in the sexual development cluster have human counterparts and that they are expressed specifically during early erythroid development. These included PF14_0774 and PFE0930w, both of which likely play roles in heme biosynthesis.

### Using automated literature mining to create new biologically relevant groups of genes

**Figure 2:**
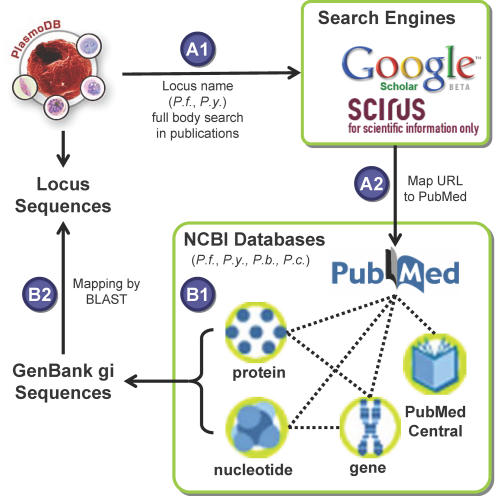
An automatic pipeline for malaria literature mining. Approach A, full text search by literature search engines: A1) All *P. falciparum* and *P. yoelii* locus names were downloaded from PlasmoDB and searched against Google Scholar and SCIRUS one at a time; A2) URL hits were then mapped to PubMed entries. Approach B, NCBI database mining: B1) Mapping between GenBank sequence entries and PubMed entries were systematically retrieved from NCBI for four *Plasmodium* species; B2) Sequences were mapped to malaria locus names by BLAST alignment. The pipeline resulted in 6,428 functional associations between 3,262 malaria proteins and 1,278 PubMed papers.

**Figure 3:**
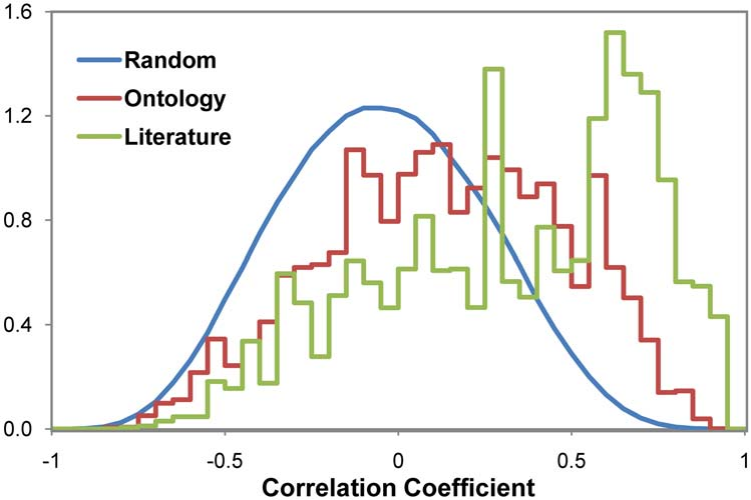
Probability density distributions of gene expression correlation coefficients. Gene pairs that either share the same ontology term (red) or are co-cited in a paper (green) are more likely to be co-expressed than randomly paired genes (blue).

Since malaria parasites have not traditionally served as model experimental systems, genome curation has mostly relied on transferring GO annotations from other model organisms via ortholog mapping. Unfortunately, this evidence-based annotation scheme is not applicable to the many parasite-specific processes that do not exist in humans or yeast. However, over the past few decades, the malaria community has investigated many *Plasmodium*-specific biological processes. In other organisms, high quality functional annotations have been assembled through automated and manual literature mining (e.g. the Saccharomyces Genome Database, http://www.yeastgenome.org). Such an approach has been adopted for model organisms, but not for *Plasmodium spp.* largely because of the low/non-profit nature of malaria research fails to justify the prohibitive cost. Therefore, we developed an automated literature-mining tool to identify groups of functionally-related malaria proteins based on their co-citation in the same manuscript or related group of publications (Figure 2). First, the World Wide Web was searched for occurrences of *P. falciparum* or *P. yoelii* locus names. An informatics pipeline was then used to process co-cited genes (Table S3, S4). Groups of co-cited genes in one manuscript or several closely related papers comprised over 1,023 virtual GO categories. A comparison in which gene expression correlation between randomly-associated genes, genes co-cited in a manuscript, or genes found in an ontology group indicated that the best correlation was amongst genes that were co-cited, no doubt because of the inclusion of expert knowledge (Figure 3). The figure shows that gene pairs within literature groups are 1,506 times more likely to have a correlation coefficient above 0.9 compared to a random gene pair. The enrichment factor for ontology groups are also as high as 62. The clear differences in the three distributions indicate genes mentioned in the same publication and genes sharing the same ontology terms are more likely to be co-regulated than by chance.

The OPI analysis was applied iteratively using this electronic knowledgebase, leading to another 58 non-redundant, statistically significant clusters (making 156 OPI clusters total when including the previous 98 clusters). Thus, groups of genes specific to parasite-specific processes such as invasion, or sporozoite development were identified (Figure 1). In many cases this approach produced better functional enrichments (*p*-value ranges from 10^−91.0^ to 10^−8.3^) than with the ontology-based method. For example, there was a high degree of co-expression among genes mentioned in a review of the process by which the parasite invades new red cells [28]. When these genes were used as seeds, the resulting OPI cluster contained 62 of the 81 *P. yoelii* or *P. falciparum* invasion genes mentioned in this review (GO:PM16497586, 134 genes, *p* = 10^−91.0^). Thus, there is a high probability that the remaining 72 uncharacterized genes in the cluster are also involved in invasion. In fact many, of these literature-driven predictions can be verified by independent protein network analysis as is discussed later.

The malaria literature database also revealed connections between proteins studied in the related apicomplexan parasite, *Toxoplasma gondii.* During invasion, apicomplexan parasites enter into a host cell by attaching to and then creating an invagination in the host cell plasma membrane. Interactions between parasite and host plasma membranes occur in the form of a ring-shaped moving junction that begins at the anterior end of the parasite and then migrates to the posterior end. The proteins are initially localized to the rhoptry neck, a subcellular organelle located at the apical end of the parasite. There were seven proteins, four from *P. yoelii* and three from *P. falciparum*, discovered in *T. gondii* that are believed to be involved in this process. All seven were found in a group of seven genes (GO:PM16244709), most of which are unannotated in *P. falciparum* [29].

### Confirmation in genetic diversity data

Previous work has shown that genes with known roles in immune evasion and host parasite interactions or which are surface-exposed have high rates of genetic variability while genes with known housekeeping function across are more conserved within and across *Plasmodium* species [7], [30], [31], [32], [33]. We also found this to be true for uncharacterized genes that are co-expressed with such characterized genes. The invasion group mentioned above (GO:PM16497586) contains some of the most polymorphic genes in the *P. falciparum* genome [32] including the merozoite surface protein 1 (PFI1475w) in which hundreds of nonsynonymous SNPs have been identified [31]. Inspection (see http://www.plasmodb.org and [31], [33]) reveals that most characterized genes (with a few exceptions) in the cluster are very polymorphic as are the uncharacterized ones. Of the uncharacterized proteins PFL2505c has at least 13 different nonsynonymous mutations across 12 *P. falciparum* strains and *P. reichenowi* relative to the sequenced strain, 3D7, PF14_0495 has 35, PF08_0035 has 16 and PFF0675c, a myosin-like protein has at least 24. In contrast, the uncharacterized proteins that are associated with genes having a known role RNA polymerase nucleolus I activity (GO:0005730, Table 2) are overwhelmingly very conserved: PF11_0471 has only one nonsynonymous mutation, PF14_0734 has one, PF08_0055 has one, PF07_0121 has one, PF11_0305 has one, PF11_0090 has four and PF10_0200 has five. This is also true for many of the other groups containing large numbers of housekeeping genes such as DNA replication clusters. Thus the genetic diversity data provides additional evidence for evaluation of possible functions.

### Independent testing using *P. knowlesi*

**Figure 4:**
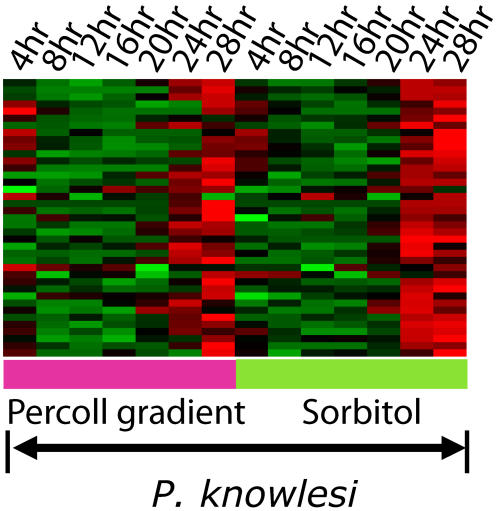
Late schizont upregulation of *P. knowlesi* genes that are orthologs of uncharacterized genes in *P. falciparum* predicted to be involved in invasion.

To empirically test our predictions we identified *P. knowlesi* orthologs of genes in the literature-derived invasion cluster. *P. knowlesi* is a simian model for human malaria and is closely related to *P. vivax*. *P. knowlesi* blood stage parasites were synchronized within the erythrocytic cycle using two independent methods and RNA was collected and hybridized to a *P. knowlesi* microarray. We expected that if the predictions from the *P. yoelii* and *P. falciparum* data were valid, we would also see upregulation of uncharacterized genes in the schizonts in *P. knowlesi*. 67 predictions were mapped to 41 unique *P. knowlesi* orthologs available on the array, almost all showed substantial upregulation (>5×) in the late schizont stage (Figure 4). The *P. knowlesi* ortholog of PFD1130w showed a 100-fold upregulation in the late schizont relative to the trophozoite stage. These data show that our predictions are likely applicable to other *Plasmodium* species as well as to other apicomplexan parasites.

### Deriving support from two-hybrid interaction data

**Figure 5:**
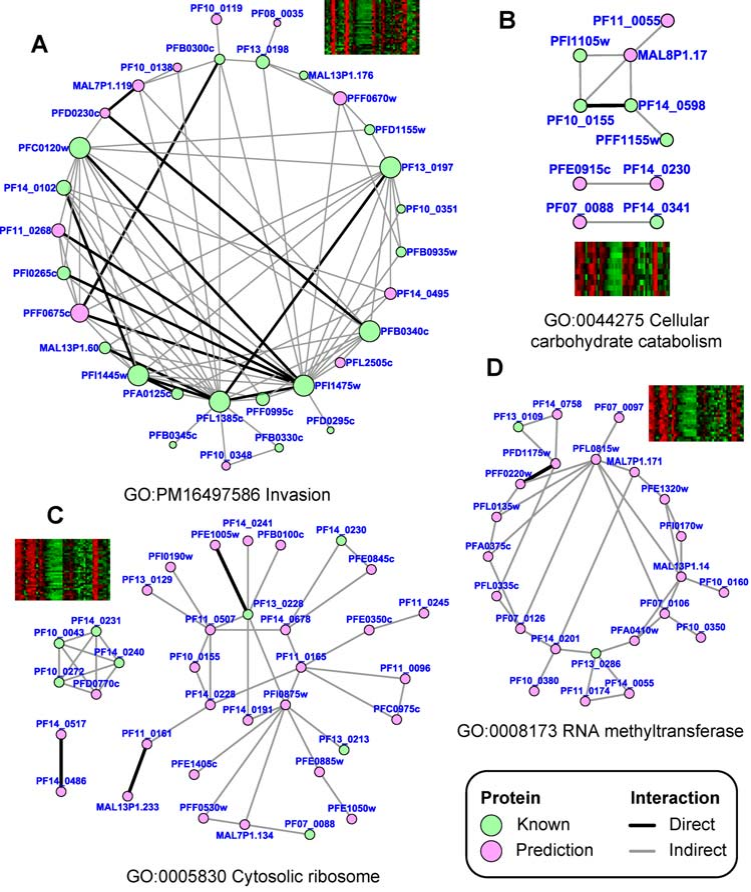
Two-hybrid interaction networks among genes that are co-expressed. A. Invasion; B. Cellular carbohydrate catabolism; C. Cytosolic ribosome; D. RNA methyltransferase activity.

Further support for expression-based function predictions were obtained from two-hybrid data from a large scale study of interactions between erythrocytic stage proteins [34]. It had previously been noted that there was enrichment for two-hybrid interactions amongst genes expressed late in the erythrocytic cycle [34]. Our new data and analysis method provided additional support for the likely significance of the two hybrid interactions in 57 clusters (*p*≤0.01) (Figure 1, Table S1). As expected, there was an abundance of interactions in the invasion cluster mentioned previously. Two hybrid interaction data were available for 35 of the 71 *P. falciparum* genes in the invasion cluster; 31 of these form 83 direct or indirect interactions pairs among themselves (Figure 5A). The probability of observing this enrichment by chance is <10^−6^. A group containing many of the genes in the cellular carbohydrate catabolism pathway (GO:0044275) also had more two-hybrid interactions than could be expected by chance alone (45 *P. f.* genes by expression, 29 proteins with two hybrid data, ten of which interact with other members of the group (*p* = 0.003)). This group included MAL8P1.17, a protein disulfide isomerase that interacts with enolase (PF10_0155), glyceraldehyde-3-phosphate dehydrogenase (PF14_0598) and phosphoglycerate kinase (PFI1105w) (Figure 5B). The cluster of 99 *P. falciparum* genes enriched for structural constituents of the ribosome had 54 genes with protein-protein interaction data within the group and 35 of these have interactions with other members of the group (*p* = 0.003) (Figure 5C). In a fourth example, we considered an expression cluster of 100 genes, several with RNA methyltransferase activity (Figure 5D). The combined two-hybrid and expression data indicated that most of the proteins in this group were very likely involved in RNA processing. For example, the uncharacterized gene PF07_0121 had a yeast ortholog factor required for a late assembly step of the 60S subunit; PF11_0305, another uncharacterized protein, had a yeast ortholog which is a constituent of the 66S pre-ribosome particle; and PF10_0200 has a yeast ortholog, YNL132w (See Table 2), which also interacts with many nucleolus ribosome assembly proteins (http://www.yeastgenome.org/, [35]). MAL13P1.14 encodes an ATP-dependent DEAD box helicase and interacts with four other members of the expression set, including PFL0815w (a DNA-binding chaperone), and two uncharacterized proteins, PF07_0106 and PFA0410w. The yeast orthologs of both MAL13P1.14 and PFL0815w are co-expressed along with other RNA modification enzymes and the yeast ortholog for MAL13P1.14 is the U3 snoRNP protein, a nucleolar protein involved in mRNA processing. Although many of the proteins were uncharacterized, this is probably because many of the genes were also uncharacterized in yeast or humans. These genes would be less likely to be good drug targets due to their cross-species conservation. The two-hybrid data includes few proteins that are expressed in the sexual or insect stages because the library was derived from blood stage material. Thus, little two-hybrid support was found for genes that have roles in sexual development, sporozoite function or other processes occurring in the non-erythrocytic stages (Figure 1). In all, protein-protein interaction analysis provided support for 970 OPI function assignments, 78% of which belong to 229 *P. falciparum* proteins not yet captured by existing manual annotations. Together with the previous cross-species co-expression evidence, we identified a total of 5,471 supported function predictions, including new functional characterizations for 926 malaria genes (Table S2).

### Upregulation of type II fatty acid biosynthesis in liver stages

One other cluster of particular interest to those interested in drug development contained a number of the genes, which were previously mentioned as upregulated in liver stage parasites relative to blood stages some of which were mentioned in a manuscript [36] describing type II fatty acid biosynthesis (GO:PM15315475, 19 genes, *p* = 10^−17.7^). Most of these genes in these clusters are expressed at very low levels in *P. falciparum* and *P. yoelii* blood stages but increase substantially in ookinetes, gametocytes and in particular in liver stages (Figure 1). The group includes PF11_0256, the pyruvate dehydrogenase alpha subunit 1; PF14_0441, the pyruvate dehydrogenase E1 beta subunit; PF08_0066, dihydrolipoamide dehydrogenase; PF10_0407, dihydrolipoamide acetyltransferase and PFF0730c, enoyl-acyl carrier reductase (ENR, the presumed target of the *Mycobacterium* tuberculosis drug, isoniazid [37] and the herbicide, triclosan) as well as their *P. yoelii* orthologs. All of these proteins have an apicoplast targeting signal and most are involved in fatty acid biosynthesis. In contrast to replication and schizogony in blood stages where a single parasite gives rise to 18 progeny, in the liver a single parasite replicates within the hepatocyte to form thousands of progeny. It is likely that there are additional needs for fatty acids that can be used to produce the membranes that will eventually surround each parasite for the thousands of new nuclei that are formed. These data suggest that fatty acid biosynthesis inhibitors may be useful against liver stage parasites. The relatively low levels of PfENR transcript in blood stage parasites relative to liver and sexual stage parasites is somewhat surprising given that inhibitors of the PfENR, such as triclosan, can cure blood stage infections in rodent parasites [38]. However, triclosan is slightly more active against parasites than against the enzyme suggesting possible off-targets effects. This may indicate that triclosan could be a more potent inhibitor of liver stage development. Given the effectiveness of the irradiated sporozoite vaccine in inducing immunity, it seems likely that drugs that cure early liver stage infections could also result in increased immunity. Furthermore, with the exception of primaquine, there are few compounds that can clear liver stage malaria infections.

It has been proposed that the protein network structure in *P. falciparum* differs from those found in other organisms [39] based on analysis of protein-protein interactions [34]. Although only 1,682 *P. falciparum* proteins have orthologs in *S. cerevisiae* or humans, proteins with orthologs are not unexpectedly concentrated in functional clusters such as mitochondrial matrix, translation, glucose metabolism, where nearly all the proteins have *S. cerevisiae* or human orthologs (Figure 1). In the group enriched for ribonucleoprotein complex (GO:0030529), 53 of the 57 genes have yeast or human orthologs. In contrast to those having to do with metabolism, genes that generally lack any type of ortholog populate clusters that contain genes involved in parasitic function such invasion or gliding motility [40]. One such cluster was enriched for genes localized to the rhoptry (combined literature, GO:PM15049814). This group contained all 11 rhoptry genes in a group of 20 (*p* = 10^−28.6^). Of these 20, none had a yeast or human orthologs. These data suggest that any small molecule targeting proteins in this cluster would be unlikely to have off-target effects. In another interesting example, a parasite-specific cluster of 65 genes contains 21 of the 30 genes that were described as sporozoite-specific (*p* = 10^−34.8^) [41]. Of the twelve proteins in the group with homologs in yeast or in humans, only succinate dehydrogenase and NAD(P)H-dependent glutamate synthase have clear cellular roles. Using annotation from a previously published analysis of expression in *P. berghei* [42], a cluster (GO:PM16908078) that contained 19 of 31 genes mentioned in the previous work was identified (61 genes, *p* = 10^−30.8^). Only 11 genes had yeast orthologs and in yeast the roles were relatively uncharacterized as well. While one possibility is that these uncharacterized genes are involved in immune evasion, or speciation, most of the genes in this study are conserved across malaria parasite species and most also have homologs in related apicomplexan parasites.

## Discussion

Existing studies have provided examples where gene functions can be correctly predicted using a “guilt-by-association” co-expression analysis, or through protein-protein interaction network analysis. Compared to co-expression analysis within a single species, identification of evolutionarily conserved cross-species co-expression patterns provides reliable functional information complementary to sequence information. Multiple species data is also much less likely to be affected by statistical randomness in the dataset or by the complexity of transcription programs [43], [44], [45]. Although cross-species expression analysis can also be applied to a few microarray experiments or sample groups [46], [47], a large panel of diversified biological conditions such as those given here provides much higher resolution and offers a level of detail that cannot be observed in smaller datasets. Since orthologous gene mapping is more likely to be a many-to-many relationship instead of one-to-one mapping, we purposefully avoided using “metagenes” [45]. Instead both *P. falciparum* and *P. yoelii* genes were retained in our expression matrix and their orthologous relationships were evaluated through unbiased statistical permutation tests. New methods have also been proposed to exploit protein-protein interaction data for functional predictions [48]. Here we use a two-hybrid data set to evaluate the statistical significance of the co-expression clusters, based on *p*-values estimated from simple node and edge counting during sampling simulations. This straightforward approach is unlikely to be controversial compared to some existing methods [49].

The comprehensive data collection presented here shows that the function of many uncharacterized proteins encoded by malaria parasites can likely be predicted based on expression patterns. To illustrate the validity of the approach we have discussed many well-characterized processes involving genes with known roles in human, parasites and yeast, as well as others where the function can be easily inferred. However, based on the accuracy of these predictions for these well-studied cellular processes such as ribosome biogenesis, it is clear that the same analysis can predict which uncharacterized proteins that are likely to have roles in less understood processes such as sporozoite function, gliding motility, or ookinete function. For example, it is very likely that the RNA binding protein PFD0825c is involved in early liver stage function given that its expression pattern mimics *pbs36*, a *P. berghei* protein critical for early liver stage development [50]. Similarly, we predict that the protein encoded by the hypothetical gene, PFI0210c, is likely involved in late stage liver development and possibly in type II fatty acid biosynthesis. The comprehensiveness of the data in this study enhances the specificity of the predictions over previous studies. While these remain only predictions, a significant portion has additional co-expression support from their yeast and human counterparts, or from independent protein network studies. It is our hope that they will serve as a basis for evaluating other types of systematic data that are produced in large-scale experiments, such as mass-spectrometric analysis of protein complexes and provide a complement to orthology mapping from model organisms. Implicit in these data is the assumption that genes that are conserved across species will be playing similar cellular roles in different malaria parasite species. Our data supports this assumption many times over, thus, if a gene is upregulated in *P. yoelii* liver stages, it seems likely that its *P. falciparum* ortholog will also be upregulated in liver stages. These data also show 549 of the uncharacterized proteins in the malaria genome participate in conserved processes that are found in other eukaryotes. Knowing whether an uncharacterized protein is likely a member of the small nucleolar complex or plays a role in pathogenesis will aid researchers in making better informed decisions. These data will also be useful for predicting the functions of genes in many related apicomplexan parasites, such as *Toxoplasma*, *Babesia* or *Cryptosporidium*. Finally, it may be that these cross-species data will be useful for understanding the function of uncharacterized genes in *S. cerevisiae* and even in humans.

## Materials and Methods

### Isolation of *P. yoelii* gametocytes

A protocol similar to that previously described [51] was used to isolate gametocytes. Three mice (strain: BALB/cByJ) were injected intraperitoneally with 0.1 ml of a 25 mg/ml stock solution of phenylhydrazine (phz) to increase reticulocytosis in the mice and increase parasitemia. The phz-treated mice were then injected with approximately 10^7^ *P. yoelii* infected erythrocytes (obtained from MR4 : *P. yoelii* 17XNL (1.1)). When the parasitemia of the mice reached ∼ 40% (after about 4 days), they were treated with sulfadiazine (15 mg/L) in their drinking water. This treatment kills asexual parasites and leaves gametocytes unaffected. The treatment was carried out for two days and mice were heart bled for the collection of the gametocytes. The blood from the mice was diluted in PBS and the leucocytes were removed by passing the blood suspension through a Plasmodipur filter (Euro-diagnostica). The gametocytes were purified from 49% Nycodenz/PBS-solution (v/v) gradient interfaces. The gametocytes found at the interface were collected and pelleted before RNA was extracted.

### Isolation of *P. yoelii* blood stages

Blood stages were isolated as previously described [52]. For schizonts blood from three infected mice (50% parasitemia) was collected and placed in complete culture medium (RPMI 1640 medium containing heat-inactivated fetal calf serum (20%), neomycin (50 IU/ml) and HEPES (25mM) containing 40 IU of heparin). The blood was incubated for 12 hours at 37°C and the schizonts were collected from the 60% Nycodenz/PBS gradient interface. These schizonts were pelleted for RNA extraction.

### Purification and preparation of total RNA from midgut and salivary gland sporozoites

The midgut and salivary gland sporozoites were obtained from infected female *Anopheles* mosquitoes on days 9 and 14 respectively post infection. The midgut and salivary gland tissues were disrupted by hand grinding and the sporozoites were isolated and counted in a hemocytometer. Sporozoite populations were purified as described previously [53]. In brief, 3 g of Diethylaminoethyl Cellulose (DE 52, Whatman) was incubated in an equilibration buffer containing 8.8 g of Tris-HCl, 5 g of NaH_2_PO_4_H_2_O, 5 g of NaCl, 18 g of glucose per liter, adjusted to pH 8.2. The preswollen DE-52 was transferred into 10 ml plastic syringe plugged with glass wool at the bottom. Ten million sporozoites were mixed with equal volume of 35% BSA and incubated on ice for 30 min. Following incubation, the sporozoites were passed through the DE-52 column. The sporozoites were eluted in 10 ml of equilibration buffer and collected as 500 µl fractions. The fractions having highest recovery of sporozoites were pooled and counted. The purification yielded 2.5–3.0 million highly purified sporozoites from each population. The sporozoites were washed twice with ice-cold PBS prior to RNA isolation. Total RNA was isolated from sporozoites using micro to midi total RNA isolation kit (Invitrogen) following manufacturer's instructions. The yields of RNA varied between 20–30 ng for each sample.

### Isolation of total RNA from *P. yoelii* infected liver

Balb/c mice were infected with 6 million *P. yoelii* sporozoites by an intravenous route. At the end of 36 hours and 40 hours post infection the mice were anesthetized and the livers were dissected out. The livers were washed two times in ice-cold PBS buffer before proceeding with the RNA extraction.

All reagents used for RNA isolation were procured from Sigma. Each liver sample was homogenized in 4 ml of denaturation buffer containing 4 M guanidium thiocyanate, 25 mM sodium citrate pH 7, 0.5% N-lauroyl sarcosine and 0.1M β-mercaptoethanol. Total RNA was extracted from six hundred microliter aliquots of each liver homogenate using the phenol chloroform method. In brief, 60 µl of 2 M sodium acetate pH 4 was added to 600 µl of liver homogenate followed by addition of 600 µl of buffer saturated phenol and 150 µl of chloroform-isoamyl alcohol. The samples were incubated on ice for 15 min and centrifuged at 10,000×g for 20 min at 4°C. Following centrifugation, 400 µl of the upper aqueous phase was transferred into a clean microfuge tube and RNA was precipitated by adding equal volumes of ice cold isopropanol. The samples were incubated at −20°C for one hour and centrifuged at 10,000×g for 15 min. The RNA pellet was washed once with cold 75% ethanol, air-dried and solubilized in 150 µl of RNAse free water. The concentration and purity of RNA were estimated spectrophotometrically.

### cDNA subtraction of liver stages

Subtraction of cDNA was performed as previously described [54]. Briefly the subtractor (uninfected mouse liver) mRNA was isolated from the total RNA using magnetic Dynabeads Oligo (dT)_25_ (Invitrogen) and directly converted to the complementary first strand cDNA, leading to immobilized cDNA on Dynabeads (DB) after melting away the mRNA. The target (mouse liver infected with *P. yoelii*) mRNA was isolated from total RNA using DB. The target mRNA was hybridized to the immobilized subtractor cDNA at 65–68°C for 20–24 hours and the common transcripts were removed by collecting the beads with cDNA/mRNA hybrids. Bead-coupled cDNA was regenerated and two more rounds of hybridization were performed using the same subtractor cDNA. After the final hybridization step, the supernatant of DB was enriched for target-specific mRNAs (*P. yoelii* liver stages). These transcripts were then captured with fresh DB and double amplified for microarray analysis.

### Isolation of *P. falciparum* zygotes

Mature gametocytes of *P. falciparum* NF54 isolate cultured *in vitro* [55]. Gametogenesis was induced by incubating mature gametocytes in exflagellation-inducing medium [56] at RT for 30 min. Zygote formation was achieved by incubation of emerged gametes in the medium for 4 hours at RT. Zygotes and gametes were enriched by centrifugation over a discontinuous 6/11/16% Nycodenz gradient and harvested from the 6–11% interface. Zygotes and gametes were washed 3 times with incomplete medium and counted in a hemocytometer. Smears were prepared, Giemsa-stained, and examined under light microscopy.

### Isolation of *P. falciparum* ookinetes

Mature gametocytes from an 18 day old gametocyte culture were diluted 1:10 with complete ookinete medium [57] supplemented with 15% FBS and incubated for 24 hours at RT with rocking. Ookinetes were purified by treatment with Lympholyte (Cedarlane Lab, Ontario, Canada), followed by centrifugation over a discontinuous 35/50/65/80% Percoll gradient [58]. Ookinetes were collected from the 35–50% interface and washed 3 times with incomplete medium, then counted in a hemocytometer. Smears were prepared, Giemsa-stained, and examined under light microscopy.

### Collection of *P. knowlesi* erythocytic expression data

*P. knowlesi* strain H1 was synchronized by two different methods, Percoll 60% [21] and Sorbitol synchronization [59]. Seven time points were collected every 4 hours for 28 hours throughout the *P. knowlesi* intra-erythrocytic cell cycle. For each time point, RNA was prepared from one 25ml culture flask with an average of 2% parasitemia and 4% hematocrit. To purify tightly synchronous schizont stages, cells were synchronized at 8 hour intervals by Percoll gradient separation for two consecutive cycles. The first time point was collected after 4 hours of incubation following the second synchronization cycle. For sorbitol synchronization, the cells were pelleted and resuspended in 5% sorbitol for 10 min, then washed and resuspended in complete RPMI media. Two successive cycles of synchronization resulted in tightly synchronous parasites. The first time point was collected after 4 hours of incubation following the second sorbitol treatment. For each time point, the cells were pelleted and resuspended in 5 volumes of Trizol. RNA was purified by Phenol extraction and purified using Qiagen RNeasy kit. RNA was amplified using the Affymetrix Genechip IVT labeling kit. 20 µg of amplified cRNA was hybridized to the Pftiling microarray for 14 hours. The gene chips were washed and scanned using standard Affymetrix protocols.

### RNA extraction and preparation of cRNA hybridizations on array

RNA extraction was performed as previously described [9]. Eight micrograms of total RNA was used for cDNA synthesis. An oligo dT primer containing a phage T7 promoter at its 5′ end was used to prime the cDNA synthesis reaction. A second strand of cDNA was then synthesized and used as a template for *in vitro* transcription in the presence of biotinylated ribonucleotide (Enzo). The labeled cRNA was then fragmented, hybridized to the array, and stained with a streptavidin phycoerythrin conjugate. Hybridizations were carried out with 15 µg of fragmented cRNA at 45°C for 16 hours, then the hybridization solution was removed and the arrays were stained and washed following Affymetrix protocols. Arrays were scanned with an emission wavelength of 560 nm at 3 µm resolution using a confocal scanner (Affymetrix), and the signal intensity for each sequence feature on the array was determined using the 70th percentile method in Microarray Suite 5 (Affymetrix).

### Double amplification methods

*P. yoelii* sporozoite RNA (initial concentration of 100ng) was subjected to a double amplification step, using a modified Eberwine protocol, to obtain eight micrograms of RNA. After the first cDNA synthesis, using an oligo dT primer containing a phage T7 promoter at its 5′ end which was utilized to prime the cDNA synthesis reaction, we used T7-in vitro transcription (Ambion MEGAscript Kit) following the manufacturer's protocol. The labeled cRNA was then fragmented, hybridized to the array, and stained with a streptavidin phycoerythrin conjugate. Hybridizations were carried out with 15 µg of fragmented cRNA, at 45°C for 16 hours, then the hybridization solution was removed and the arrays were stained and washed following the Affymetrix protocols. Arrays were scanned with an emission wavelength of 560nm at 3 µm resolution using a confocal scanner (Affymetrix), and the signal intensity for each sequence feature on the array was determined using the 70th percentile.

### Malaria gene expression datasets

The TSRI Malaria custom array (scrMalaria) contains probes for 5,159 *P. falciparum* genes and 5,521 *P. yoelii* genes. The *P. yoelii* probes were designed to hybridize to predicted genes from *P. yoelii* 17XNL line clone 1.1 genome sequence [5]. The array was used previously to systematically profile the *P. falciparum* life cycle. Previous studies included the hybridization of 38 *P. falciparum* samples, including erythrocyte stages, detailed gametocyte time courses, and one sporozoite stage [9], [60]. This study adds 2 *P. falciparum* samples, including zygote and ookinete stages, and 14 *P. yoelii* samples, including three blood stages (one schizont and two mixed stages), four gametocyte samples, five sporozoite and two liver stage (36 and 40 hours post infection). As with the *P. falciparum* design, there are a variable number of probes per gene and thus higher confidence data is obtained for longer genes. All array measurements were processed using match-only integral distribution (MOID) algorithm as described previously [60]. A quantile normalization method [61] was then applied to the *P. yoelii* and *P. falciparum* data independently, in order to minimize the variation that resulted from different hybridizations (collected over a five year period) having different dynamic ranges. Genes with six or more probes, maximum expression level of 50 units in one sample, a fold change greater than two within a dataset were considered non-trivial for correlation calculations. This includes 3,690 *P. falciparum* genes and 2,902 *P. yoelii* genes. All malaria expression data are available through our web site (http://carrier.gnf.org/publications/Py) and PlasmoDB (http://www.plasmoDB.org).

### Yeast gene expression datasets

The yeast expression dataset consists of 74 samples accumulated in GNF's in-house yeast gene chip database, many of which have not been published before. The set includes data from 62 yeast cell cultures that had been treated with small molecules (latrunculin, itraconazole, ketoconazole, etc.), exposed to different growth conditions (UV treatment, heat shock, cold shock, low salt, high salt media) [62], [63], or synchronized within the cell cycle (sampled every 10 min). All samples were profiled using the Affymetrix S98 array that consists of 6,135 yeast genes. All array measurements were processed and normalized using Affymetrix MAS4 algorithm, 4,515 unique gene entries with expression fold change greater than 1.5 and standard deviation greater than 40 were retained. This dataset, as well as detailed sample descripftions, is available through our web site (http://carrier.gnf.org/publications/Py).

### Human gene expression datasets

The human tissue expression dataset is distributed by GNF's SymAtlas web site (http://symatlas.gnf.org) [25]. This dataset consists of 79 human tissues in duplicates, measured using Affymetrix U133A array that consists of 22,215 probe sets. All array measurements were processed and normalized using Affymetrix MAS5 algorithm; 13,499 unique gene entries with expression level both above noise and a fold change greater than 1.5 across all tissues were retained.

### Ortholog pairing and malaria expression extrapolation

Orthologous groups of proteins defined by OrthoMCL database (version 2.0) were used in this study [64]. OrthoMCL maps proteins across multiple species, including *P. falciparum*, *P. yoelii*, *P. berghei*, *P. chabaudi*, *S. cerevisiae*, *H. sapiens*, etc. Within each ortholog group, all *P. falciparum* protein sequences and *P. yoelii* protein sequences pairs are first pair-wise aligned using BLAST. Then for a given *P. falciparum* protein, the *P. yoelii* ortholog that can be found in the malaria array (≥6 probes) and provides the best alignment score is selected as the best match. The same matching process was applied to identify the best *P. falciparum* match for each *P. yoelii* protein. We also took into account 3,969 best reciprocal BLAST match pairs between the two species identified using E-value≤10^−2^, as well as additional sequence alignment pairs of E-value≤10^−6^. Best orthologs were assigned to 4,469 *P. falciparum* genes and 5,258 *P. yoelii* genes.

We aimed to construct a mostly complete malaria expression matrix, i.e., the rows comprise all the differentially expressed *P. falciparum* and *P. yoelii* genes, and the columns are the combination of all parasite life stages profiled in either species (54 samples in total). For each *P. falciparum* gene, its expression values in the 14 *P. yoelii* samples were extrapolated by copying the expression vector of its *P. yoelii* best ortholog match. For each *P. yoelii* gene, its expression values in the 40 *P. falciparum* samples were copied from the expression vector of its *P. falciparum* best ortholog match. The expression extrapolation process resulted in a final matrix of 6,592 malaria genes across all 54 parasite samples. Notice that a reciprocal best match protein pairs will lead to two identical expression vectors, therefore such gene expression dependency will need to be carefully modeled in the OPI permutation testing described later.

### Derivation of P. yoelii Gene Ontology database

Gene annotations for *P. falciparum* were downloaded from Gene Ontology consortium (http://www.geneontology.org). Additional annotations were downloaded from PlasmoDB (http://www.plasmodb.org) and combined. GO assignments for *P. yoelii* genes were derived by inheriting all GO terms assigned to its *P. falciparum* orthologs, which were defined according to OrthoMCL database. As the result 3,763 *P. yoelii* genes have assignments in 2,889 GO categories.

### Protein network analysis

Systematic protein-protein interaction dataset for *P. falciparum* was made available by a previous study [34]. Protein network analysis described below was applied to the *P. falciparum* proteins within each of the 156 OPI clusters. For any given cluster of *N*_T_ *P. f.* proteins, the subset of entries that form either direct or indirect protein-protein interaction pairs among themselves was identified. An indirect interaction was defined as two proteins that interact and only interact via at least a third protein, similar to a previous network study [39]. We focused on two characteristics of each resultant network, *i.e*., its node count *N*_N_ and edge count *N*_E_, where each node uniquely represents a protein and each edge represents a unique interaction pair. We then randomly sampled *N*_T_ number of proteins from the total collection of 3,691 *P. f*. proteins comprising the expression matrix described above, and counted the nodes and edges in the corresponding resultant network. Such simulations were repeated at least 1000 times, and the percentage of runs that led to either a higher node count than *N*_N_ or a higher edge count than *N*_E_ gave an unbiased estimation of the *p*-value. Protein networks with a *p*-value≤0.01 are considered to show significant connectivity (Figure 1, Table S1). For such cases, protein network data provide an additional piece of evidence for the correct functional assignment based on expression clustering (Table S2). All statistically significant protein networks were plotted graphically and made available on our web site, together with the human and yeast orthologous evidences.

### An automatic pipeline for malaria literature mining

To collect malaria functional annotations from existing literatures, we took two complementary approaches. In approach A, *P. falciparum* and *P. yoelii* locus names downloaded from PlasmoDB were used as search terms one at a time through Google Scholar beta (http://scholar.google.com) and SCIRUS (http://www.scirus.com) search engines. Gene/protein names or description keywords were not used in our searches, because of their high number of false positives. Not limited by the title and abstract stored in the NCBI PubMed database, both Google Scholar and SCIRUS index the full literature text, and therefore are expected to provide complementary annotations to what already exists in NCBI. Google Scholar database mostly consists of manuscripts accessible publicly via World Wide Web, while SCIRUS collects additional non-publicly accessible literature; including some essential Elsevier journals, which frequently publish research results from malaria scientific community. Each search returned a list of URLs (we kept a maximum 500) and some association summary data such as paper title, journal name, volume, and start page, etc. As multiple URLs could refer to the same publication, we mapped every URL hit by an automatic query to the PubMed database and translated the URL into a PubMed identifier whenever possible. In this way, proteins co-cited in the same paper were discovered even though they were obtained from independent searches. Developing efficient search robots in this approach was difficult, mainly due to the lack of programming interface from both search engines. We therefore had to limit our searches to *P. falciparum* and *P. yoelii* only. Searches with *P. falciparum* systematic names resulted in 337 papers and searches with the *P. yoelii* names only identified an additional 15 new papers, therefore, we anticipate relatively few papers exist that cite *P. berghei* and *P. chabaudi* genes by their locus names.

When publications are related to genome sequencing projects, reports of new technology development for malaria systems biology, or other studies irrelevant to specific parasite functional characterization, they tend to contain large number of locus identifiers but are not interesting to our study. We manually filtered out 26 such papers. In total, we identified 2,605 associations between 1,509 malaria proteins and 326 PubMed papers through approach A.

In Approach B, we first downloaded all GenBank sequences for *P. falciparum*, *P. yoelii*, *P. berghei* and *P. chabaudi* that have associations with PubMed entries. The association can be either a direct link or sometimes an indirect link derived through its link first to an NCBI Gene database entry then to PubMed database entries. A unique GenBank gi number identifies each sequence. Genomic sequence entries such as chromosome fragments or genome shotgun sequences were filtered out. Extremely long or short sequences were also discarded, so that only nucleotide sequences of length between nine and 60,000 bases and proteins sequences with lengths between three and 20,000 amino acids were retained. All the data were retrieved through NCBI eUtils programming interface. We then downloaded the complete transcriptome and proteome sequences of all four Plasmodium species from PlasmoDB, where sequences are identified by their locus names. Next, a BLAST search was conducted for each gi sequence against the corresponding transcriptome or proteome within the same species with an E-value threshold of 10^−6^. Given either a protein sequence alignment of at least 90% identity over 50 amino acids or a nucleotide sequence alignment of minimal 95% identity over 100 nucleotide bases, a gi number was mapped to a locus name whenever possible. Once matched, all PubMed entries associated with the gi number were automatically transferred to the locus entry. After filtering out 18 less relevant publications, we identified 3,901 associations between 2,350 malaria proteins and 1,006 PubMed papers through this approach.

Combining both approaches into an automatic literature-mining pipeline resulted in a total of 6,428 associations between 3,262 proteins/genes and 1,278 papers. The complete literature mining results are available in a searchable database on our website http://carrier.gnf.org/publications/Py. (follow the “literature database” link, also Table S3, S4).

### Derivation of literature-based malaria annotations

Our literature-mining pipeline produced custom annotations in the form of malaria protein and PubMed paper associations. For a given paper, we expand the malaria protein list by including all orthologs across four Plasmodium species as defined by OrthoMCL database. Each paper was treated as its own virtual GO category and all the malaria proteins associated were considered as members of that category. Gene Ontology database consists of three branches, i.e., biological process, molecular function, and cellular component. These literature-derived virtual GO categories did not undergo the same curation process as real GO categories and therefore best kept under a separate “literature” branch to avoid misinterpretation. Otherwise, these virtual GO categories were treated the same by the later OPI clustering algorithm. We also carried out limited manual curation in order to merge proteins of some closely related papers into a newer virtual category. The rationale is such combination typically produces more complete annotation categories and should lead to better sensitivity in the clustering analysis.

### Annotation-driven OPI clustering analyses

OPI is a supervised clustering algorithm that produces the highest statistical enrichment for the known genes in an optimal co-expression cluster of a given GO category. The details of OPI algorithm, its comparison with other clustering algorithms, as well as its applications to the *P. falciparum* global gene expression analysis were described previously [8], [60]. One may also find many applications of a similar supervised clustering algorithm called Gene Set Enrichment Analysis (GSEA) [65], however, OPI is more suitable for function prediction of uncharacterized genes. In this study, clustering analysis was independently applied to the three gene expression datasets mentioned above. First, genes that did not show significant expression variation were filtered out. Then, the program iterates over all gene ontology groups containing a minimum of two known genes in the expression data matrix; this corresponds to 2,708 GO groups (including our virtual literature-derived categories) for the malaria dataset, 2,210 GO groups for the yeast dataset, and 4,428 GO groups for the human tissue dataset.

Expression vectors of genes in the expression matrix are not independent and the various categories in the GO tree are also correlated, therefore the optimal hypergeometric *p*-value produced by OPI algorithm will need to be double checked by randomly generated dataset so that dependency are modeled correctly. For yeast and human dataset, randomization testing was carried out straightforwardly by shuffling gene labels in the expression matrix as described previously [60]. Permutation schema for malaria dataset, however, requires extra steps. This is because the expression extrapolation step made use of ortholog mapping information and introduced additional coupling between expression vectors. *P. falciparum* and *P. yoelii* genes were first randomly relabeled among themselves, respectively. The whole expression extrapolation process used to produce the original combined malaria dataset was then repeated, so that the ortholog correlation is retained in the resultant randomized datasets as well. For each dataset we repeated OPI analyses on 100 independently randomized expression matrix. An original OPI cluster is considered truly statistical significant only if equal or better *p*-value can be achieved by no more than 5% of the permutation runs. The percentage of genes not already annotated under the given GO category in an OPI cluster estimates the false discovery rate (FDR) of co-expression-based function prediction. OPI clusters with size greater than 500 are often associated with relatively high false discovery rate and are unsuitable for the sole purpose of gene function prediction; these clusters were filtered out despite the fact that they may still be justified by pure statistical significance. The final results of the OPI analyses included 684 clusters for yeast and 1,047 clusters for human. The list of known genes in different ontology groups can be sometimes identical or very similar, resulting in closely related OPI clusters. To ease the analysis, we only kept a total of 156 clusters for malaria dataset, where such redundancy has been removed. Among them, 58 clusters were derived from literature-based custom GO groups. If a biological process of the parasite is conserved in yeast or human, cross-species comparison of related OPI clusters enables us to find additional evidence to support newly predicted *Plasmodium* genes functions. All clusters for the three datasets and ortholog evidences are accessible on our web site (http://carrier.gnf.org/publications/Py).

### Unsupervised clustering of distinctly expressed malaria genes

For all 6,592 non-trivially expressed *P. falciparum* and *P. yoelii* genes used for OPI analysis, 4,712 genes are members of the 156 OPI clusters, another 736 genes fall into clusters that are statistically significant (permutation *p*≤0.05), but their function predictions are not presented due to the large cluster size, and another 118 genes share similar expression profiles (Pearson correlation coefficient ≥0.8) with at least one statistically significant OPI cluster. The remaining 1026 genes do not share expression pattern with sufficient number of previous characterized genes. The hierarchical clustering algorithm was applied to each subset of genes and the results are accessible on our website (http://carrier.gnf.org/publications/Py).

### Probability density distributions

For the 3,690 *P. falciparum* genes used in the OPI analysis, 1,037 GO groups and 514 literature groups were found to contain 2 to 100 genes. The probability density distribution of two randomly selected genes was calculated by using all the 6,806,205 possible gene pairs. For the literature and GO distributions, Pearson correlation coefficients were first calculated for all gene pairs within a group. The coefficients were then weighted, such that each group contributed equally to avoid bias caused by large groups. In total, 239,577 and 139,811 gene pairs were used to derive the GO distribution and literature distribution, respectively.

## Supporting Information

Table S1 The list of 156 statistically significant OPI clusters, their ortholog counts in *S. cerevisiae* and human, and their *P. falciparum* protein counts that form within-cluster networks.
(0.08 MB XLS)

Table S2 Yeast and human orthologs evidence and protein interaction evidence for assigned and predicted gene ontology memberships of malaria genes.
(3.40 MB XLS)

Table S3 The complete list of malaria-related PubMed publications.
(3.20 MB XLS)

Table S4 The complete list of malaria genes cited in each malaria publication.
(0.75 MB XLS)
